# Manipulating early-life environment and handling alters growth trajectories, immune and stress responses, clinical indicators, and intestinal health in young pigs

**DOI:** 10.1093/jas/skaf343

**Published:** 2025-10-06

**Authors:** Kaitlyn M Sommer, Zimu Li, Ryan N Dilger

**Affiliations:** Department of Animal Sciences, University of Illinois, Urbana, IL, 61801, USA; Neuroscience Program, University of Illinois, Urbana, IL, 61801, USA; Department of Animal Sciences, University of Illinois, Urbana, IL, 61801, USA; Neuroscience Program, University of Illinois, Urbana, IL, 61801, USA

**Keywords:** handling, immune response, pigs, rearing environment, stress

## Abstract

Stressors are factors that disrupt homeostasis; in pigs this includes challenges in sow management, human interaction, and weaning. These typically occur in the first month of life, compromising welfare and decreasing growth and increasing mortality. This study investigated the impact of early-life stressors on pig growth, immune response, and coping ability. The study utilized a 2 × 2 factorial arrangement with factors including rearing environment [2 levels; sow-reared (SR) or artificially-reared (AR)] and handling (2 levels; handled for 2 min daily or weekly). Therefore, 72 pigs (38 gilts and 34 boars) were allotted to 1 of 4 treatments on postnatal day (PND) 2 based on their litter of origin and body weight (BW): 1) SR-W (sow-reared, weekly-handled); 2) SR-D (sow-reared, daily-handled); 3) AR-W (artificially-reared, weekly-handed); and 4) AR-D (artificially-reared, daily-handled). AR pigs were housed individually with ad libitum access to reconstituted milk replacer, while SR pigs were group housed and received all nutrients from the sow. On PND 21, rearing environment and handling interventions ended, and pigs were transferred to group nursery pens according to their original treatment assignment. One week later, on PND 28, all pigs received a 5ug/kg of BW injection of lipopolysaccharide to stimulate an innate immune response. On PND 35, all pigs were euthanized to permit sample collection. Data were analyzed using a 2-way ANOVA via the MIXED procedure of SAS. During PND 2-21, AR pigs had greater (*P* < 0.05) BW gain than SR pigs. Consequently, AR pigs had the heaviest (*P* < 0.05) BW on PND 21 and 35 compared with SR pigs. However, SR pigs exhibited higher (*P* < 0.05) feed efficiency during PND 21-35 compared with AR pigs. As a classic marker of stress, fecal secretory immunoglobulin A concentrations were highest (*P* < 0.05) in SR pigs compared with AR pigs on PND 21. On PND 35, daily-handled pigs exhibited longer (*P* < 0.05) small intestinal tract length than weekly-handled pigs. Furthermore, SR pigs had increased (*P* < 0.05) absolute and relative ileal weights, but lower (*P* < 0.05) absolute duodenal mass compared with AR pigs. Lastly, daily-handled pigs displayed increased (*P* < 0.05) TNF-α, IL-1β, and occludin mRNA expression relative to weekly-handled pigs, while AR pigs had increased (*P* < 0.05) mRNA expression of IFN-γ and IL-1β compared with SR pigs. In conclusion, early-life rearing environment and handling frequency influenced growth, immune and stress response, clinical indicators, and intestinal health.

## Introduction

Advancements in genetic selection and farm management practices over the past decades have contributed to an increase in sow litter sizes. However, this rise in piglets per sow can eclipse the number of teats available for nursing (Baxter et al., 2013; Zhang et al., 2021; Han et al., 2024). An inability for a newborn pig to claim a teat can lead to limited colostrum intake, compromising passive immunity and heightening the risk of exposure to novel pathogens while the immune system is still developing (Le Dividich et al., 2005). A prolonged inability to claim a teat can exacerbate susceptibility to physiological stress due to hypothermia or undernutrition, as well as psychological stress due to intensified competition and aggression among littermates. In severe cases, this can result in increased morbidity and mortality due to limited growth and overt malnutrition (Le Dividich et al., 2005; Zhang et al., 2021). To balance litter sizes, ensure an appropriate piglet-to-teat ratio, and achieve uniform body weights at weaning, producers may employ strategies such as cross fostering (Robert and Martineau, 2001) or artificial rearing. These interventions typically occur after the first 48 h after farrowing to guarantee initial colostrum intake (Robert and Martineau, 2001; Schmitt et al., 2019a). Cross fostering involves transferring pigs from their birth sow to a recipient sow that has available teats to provide timely maternal care (Zhang et al., 2021; Byrd et al., 2022). In contrast, artificial rearing removes pigs from the sow entirely and places them in separate housing where they are either individually or group-housed (Braude et al., 1970; Schmitt et al., 2019b).

Artificial rearing provides essential resources to support pig growth, health, and well-being and this rearing context has been observed to reduce mortality, morbidity, and growth limitations in vulnerable pigs (Schmitt et al., 2019a; Han et al., 2022; Sommer et al., 2025). Additionally, it offers precise control over milk provision, including timing and volume of liquid nutrition, while enabling the assessment of pig growth performance through metrics such as milk disappearance (Sommer et al., 2025). However, the close monitoring of growth performance and natural consumption patterns in an artificial rearing environment may induce stress due to frequent human interaction and handling for BW assessments (Fil et al., 2021; Sommer et al., 2025). Moreover, artificial rearing should be regarded as a form of early weaning, as young pigs experience psychological stress upon separation from the sow (Schmitt et al., 2019a). Furthermore, exposure to psychological stress can trigger the hypothalamic-pituitary-adrenal axis, leading to the synthesis of glucocorticoids, primarily cortisol. Elevated systemic cortisol levels can disrupt the microbiota-gut-brain axis, potentially resulting in intestinal dysbiosis (Frese et al., 2015; Nowland et al., 2022). Such a shift in microbial composition may subsequently impair mucosal homeostasis, which can activate the immune system. This context can also promote pathogen overgrowth, contributing to the pathogenesis of intestinal disorders and further modulate the pig’s immunity (Greese et al., 2017; Li et al., 2017; Wang et al., 2019). Despite these challenges, early-life exposure to the combined stressors of early weaning and routine handling may enhance a pig’s ability to cope with stress, potentially influencing their adaptation to dry diets and interactions with novel conspecifics in subsequent growth phases.

Weaning is a critical developmental stage for pigs, marked by nutritional, physiological, and psychological stressors (Pluske and Williams, 1996). Under typical conditions in the commercial U.S. swine industry, weaning occurs between 18 and 28 days of age, at which point young pigs are separated from the dam and introduced to novel diets, environments, conspecifics, and potential pathogens (Campbell et al., 2013; Rhouma et al., 2017; van Kerschaver et al., 2023). This abrupt dietary transition often triggers a period of anorexia leading to concurrent growth reductions and structural and functional changes in the small intestine (Lalles et al., 2009; Campbell et al., 2013; Rhouma et al., 2017; Pluske et al., 2018a). Research suggests that the younger a pig is when weaned, the greater the likelihood of experiencing growth and intestinal perturbations (Carroll et al., 1998; Moeser et al., 2007;). However, limited studies have examined whether stress from early weaning and increased handling enable pigs to develop coping mechanisms that mitigate the stress response at weaning, particularly when compared with pigs reared in a traditional context (ie, on the sow). Therefore, the objective of this study was to assess the impact of pre-weaning environment and handling on pre- and post-weaning growth, stress, and immune responses. We hypothesized that artificially reared, daily-handled pigs would demonstrate improved growth performance and adapt more rapidly to group housing and complex dry diets, reflecting increased resilience compared with less frequently handled, sow-reared pigs.

## Materials and Methods

All animal care and experimental procedures were approved by the University of Illinois Institutional Animal Care and Use Committee prior to study initiation.

### Animal husbandry and experimental design

A total of 10 sows were sourced from the Swine Research Center at the University of Illinois Urbana-Champaign (PIC Camborough gilts × PIC 800 boars) 9–10 days prior to farrowing and transported to the Veterinary Medicine Research Farm. The day prior to the expected common farrowing date, sows received an intramuscular injection of 0.7 mL of cloprostenol (Estrumate, 250 µg/mL; Merck Animal Health, Rahway, New Jersey) to induce farrowing. All pigs were allowed to suckle for up to 48 h after birth to ensure colostrum intake. Approximately 48 h after the last litter was born, piglets received a 2-mL dose of *Clostridum perfringens* antitoxin C and D (Colorado Serum Company, Denver, CO) subcutaneously and a 2-mL dose orally, along with a 200 mg intramuscular dose of iron dextran (Uniferon 200, Pharmacosmas, Inc., Wachung, NJ) to prevent iron deficiency. At the same time, pigs were administered 0.25 mL of ceftiofur (Excede; Zoetis inc. Parsippany, New Jersey) intramuscularly as a prophylactic measure aligned with the University of Illinois veterinary standard of care. Following injections, pigs were assigned to treatment groups according to their body weight and litter of origin.

This study utilized a 2 × 2 factorial treatment arrangement, with rearing environment (sow-reared or artificially-reared) and handling (2 min of individual handling either daily or weekly) as the main factors. Therefore, pigs were assigned to one of four treatments: 1) SR-W (sow-reared, weekly-handled; *n* = 18); 2) SR-D (sow-reared, daily-handled; *n* = 20); 3) AR-W (artificially-reared, weekly-handed; *n* = 16); and 4) AR-D (artificially-reared, daily-handled; *n* = 18). Rearing environment and handling frequency interventions occurred from postnatal day (PND) 2-21 and ceased once pigs were transitioned to the group-housing context. This study was conducted in 2 cohorts, utilizing a total of 72 pigs (38 gilts and 34 boars). Based on group pens, treatment replications were uneven within cohort, so cohort was included as a random variable within the statistical model. Litters were not standardized as a subset of pigs from each litter was removed from each dam after allotment and reared artificially. This resulted in each sow having 4–10 suckling pigs remaining, which was assumed to limit competition for nutrients among the pigs remaining with the dam.

### Pre-weaning rearing environment

Pigs in sow-reared treatments remained with their respective dam and littermates in farrowing crates (2.03 m long × 0.61 m wide) until 21 days of age (Fil et al., 2021). The rearing environment was maintained at 22 °C with a 12 h light cycle (0800 h to 2000 h). Sow-reared pigs received all nutrients from their birth dam (ie, no cross fostering occurred) as no supplemental water, milk replacer, or creep feed were provided. Body weight was recorded either daily or weekly depending on the assigned handling treatment. Daily health checks were performed for all pigs to track lethargy, weight loss, vomiting, and fecal consistency as clinical health indicators.

Pigs in artificially-reared treatments were transferred to the Piglet Nutrition and Cognition Laboratory at the University of Illinois at Urbana-Champaign on PND 2. Pigs were housed in stainless steel cages (1.03 m deep × 0.77 m wide × 0.81 m high) with clear, acrylic facades and side walls bearing many small openings (2.54 cm in diameter) to allow for adequate ventilation (Fil et al., 2021). The caging design ensured that pigs could smell, hear, and see, but not directly touch, neighboring conspecifics (Fil et al., 2021). Ambient room temperature was maintained between 27–29°C. A 12 h light/dark cycle was maintained with light from 0800 h to 2000 h daily. For the first 5 d, pigs received an electrolyte solution (Swine BlueLite; TechMix, Stewart, MN) due to typical incidence of loose stool in AR pigs. A standard commercial milk replacer (Multi Species Milk Replacer; Land O Lakes, Arden Hills, MN; **Table 1**) was reconstituted fresh daily at 200 g of dry powder per 800 g of tap water. Milk replacer was provided using an automated liquid delivery system from 1000 h one day to 0600 h the next day. Any milk replacer remaining at the end of each feeding period was subtracted from the initial volume to quantify milk disappearance over the 20-h feeding period, referred to hereafter as intake. Water was offered ad libitum via a cup drinker. Daily health checks were performed for all pigs to track lethargy, weight loss, vomiting, and fecal consistency as clinical health indicators.

**Table 1.** Calculated nutritional composition of milk replacer^1^

Nutrient	Minimum	Maximum
**Crude protein^2^, %**	24.00	–
**Crude fat, %**	24.00	–
**Crude fiber, %**	–	0.15
**Ash, %**	–	10
**Lysine, %**	2.10	–
**Calcium, %**	0.75	1.25
**Phosphorus, %**	0.80	–
**Sodium, %**	–	0.85
**Copper, mg/kg**	6	11
**Vitamin A, IU/kg**	66,138	–
**Vitamin D_3_, IU/kg**	11,023	–
**Vitamin E, IU/kg**	253.5	–
**Vitamin B_12_, mg/kg**	44.1	–
**Ascorbic acid, mg/kg**	441.0	–

1 Guaranteed nutrient analysis (Multi Species Milk Replacer; Land O Lakes, Arden Hills, MN).

2 *N* × 6.25.

### Post-weaning rearing environment

On PND 21, rearing and handling interventions ceased, and all pigs were moved to age-appropriate, raised deck nursery pens (1.35 m × 1.50 m; vinyl coated, expanded metal flooring), with a nipple drinker and 4-hole feeder per pen where they were group housed through PND 35. Pigs were assigned to pens per previous treatment allocation and each treatment was represented by a minimum of 3 mixed-sex pens containing 4–8 pigs. Pigs had ad libitum access to water and common, age-appropriate diets in each of 3 consecutive feeding phases **(****Table 2****)**. Feeders were weighed weekly to calculate pen intake. Once group housed, pigs were no longer handled based on assigned treatment, but all pigs received incidental handling as required for blood collection and weighing events.

**Table 2.** Ingredient composition of nursery diet^1^

Item	Value
***Ingredient, %***	
** Corn, yellow dent, ground**	42.52
** Soybean meal, 48% crude protein**	22.00
** Whey, dried**	25.00
** Spray-dried porcine plasma^2^**	4.75
** Choice white grease**	2.00
** Limestone**	1.35
** Dicalcium phosphate**	0.45
** Salt**	0.20
** Mineral premix^3^**	0.35
** Vitamin premix^4^**	0.20
** L-Lysine HCL**	0.35
** DL-Methionine**	0.15
** L-Threonine**	0.08
** Zinc oxide**	0.40
** Chlortetracycline^5^**	0.20
***Calculated Composition^6^***	
** ME, kcal/kg**	3,375
** CP, g/kg**	205.9
** Ca, g/kg**	8.5
** Total P, g/kg**	5.8
**SID amino acids, g/kg^6^**	
** Lysine**	14.3
** Methionine + cysteine**	7.8
** Tryptophan**	2.6
** Threonine**	8.5
** Valine**	9.1

1 Diets were formulated for nursery-aged pigs (21–35 d).

2 Appetein (APC, Ankeny, IA) granulated plasma.

3 Trace mineral salt (ADM, Decatur, IL) included the following per kg of complete diet: salt maximum, 70 g; iron (ferrous sulfate), 51.4 mg; zinc (zinc oxide), 57.2 mg; manganese (manganous oxide), 20 mg; copper (copper sulfate), 8 mg; iodine (calcium iodate), 0.35 mg; selenium (sodium selenite), 3 mg.

4 Vitamin premix (ADM, Decatur IL) included the following per kg of complete diet: vitamin A (retinyl acetate), 6,600; vitamin D_3_ (cholecalciferol), 660 IU; vitamin E (dl-α -tocopherol acetate) , 88 IU; vitamin K (menadione sodium bisulfite), 4.4 mg; vitamin B12, 0.04 mg; riboflavin, 8.81 mg; d-Pantothenic acid (calcium pantothenate), 24.05 mg; niacin (niacinamide), 33.07 mg; choline (choline chloride), 286.38 mg.

5 Aureomycin 100 (Zoetis, Troy Hills, NJ).

6 Metabolizable energy and standardized ileal digestibility amino acid values were calculated using NRC (2012). Analyzed crude protein determined as CP = (*N* × 6.25).

Abbreviations: CP, crude protein; ME, metabolizable energy; SID, standardized ileal digestibility

### Innate immune stimulation

On PND 28, pigs were administered 5 µg of reconstituted lipopolysaccharide (LPS; *E. coli* strain O111: B4, Sigma Aldrich, St Louis, MO) per kg of BW via a single intraperitoneal injection. LPS was utilized as a component of gram-negative bacterial cell walls to stimulate a transient inflammatory response, thereby inducing an acute innate immune reaction, serving as a mimetic of an acute, inflammatory challenge that pigs may experience during the nursery phase. The dosage utilized was selected as previous research has reported is ability to activate the acute phase response in pigs (Johnson and von Borell, 1994; Webel et al., 1997; Moya et al., 2008).

### Data and sample collection

**Figure 1.**
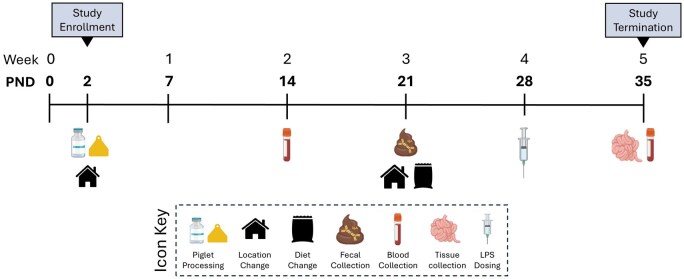
Study timeline depicting all study outcomes and time-points except for days when body weight was recorded. Dependent on treatment, pigs handled daily were weighed during each handling event for the first 3 weeks of study. On PND 21, handling frequency and rearing environment interventions ceased and pigs were handled weekly for body weight measurements. Abbreviations: LPS, lipopolysaccharide; PND, postnatal day. Created using BioRender.com.

Study outcomes followed the timeline displayed in **Figure 1**.

### Growth performance

From PND 2-21, BW was recorded dependent on handling treatment (daily or weekly) and BW was used to calculate average daily gain (ADG). For pigs that were artificially reared, average daily feed intake (ADFI) and gain-to-feed ratio (G: F) were also measured. During the PND 21-35 period, pigs were individually weighed weekly, and feeder weights were recorded to calculate ADG, ADFI, and G: F while in the group context.

### Sample collection

On PND 14, pigs underwent a jugular blood draw to permit analysis of clinical chemistry and hematology outcomes and samples were submitted to the University of Illinois at Urbana-Champaign Veterinary Diagnostic Laboratory. Blood serum was collected for clinical chemistry and allowed to clot for up to 45 min before being placed on ice. Blood plasma was collected and immediately placed on ice before submission. Representative fecal samples were collected on PND 21 and 27, snap frozen in liquid nitrogen, and then stored at -80°C until analysis.

The study ended on PND 35 and pigs were anesthetized via an intramuscular injection of telazol: ketamine: xylazine solution [50 mg of zolazepam reconstituted with 2.50 mL or ketamine (100 g/L) and 2.5 mL xylazine (100 g/L); Fort Dodge Animal Health, Overland Park, KS] at 0.08 mL/kg BW. After sedation, blood was collected via cardiac puncture for submission of samples to the University of Illinois at Urbana-Champaign Veterinary Diagnostic Laboratory. Plasma tubes were placed on ice for hematology, and serum tubes were allowed to clot for up to 45 min before being placed on ice for clinical chemistry determination. Once blood collection was completed, pigs were euthanized using sodium pentobarbital (Euthasol Euthanasia Solution CIIIN; Patterson Veterinary Supply, St Paul, MN) at 1 mL per 4.5 kg of BW via cardiac injection. Subsequently, the small intestine was dissected from the pyloric sphincter to the ileocecal valve for subsequent measurements and tissue collection.

### Fecal secretory IgA

Fecal samples collected on PND 21 and 27 were thawed and aliquoted separately for dry matter and secretory immunoglobulin A (IgA) analyses. Dry matter was determined after drying sub-samples in a 105 °C oven for a minimum of 48 h (method 934.01, AOAC Official Method of Analysis, 2000). A porcine-specific enzyme-linked immunosorbent assay (ELISA) was used to analyze fecal secretory IgA, with 0.1 g of sample aliquoted into snap-cap tubes before adding 1 mL of phosphate buffered saline. Subsequently, the sample tubes were homogenized (TissueLyser II, Qiagen, Valencia, CA) at 30 Hz for 15 sec before being centrifuged for 15 min at 12,000 × g. The upper aqueous layer was collected and utilized to quantify secretory IgA concentrations via a sandwich ELISA (catalog # PGA51-01; Eagle biosciences; Nashua, NH). Secretory IgA concentrations were analyzed with an analytical sensitivity of 4.380 ng/mL, standard curve range of 0–400 ng/mL, and inter- and intra-assay CV each less than 10%.

### Small intestinal organ metrics

After extraction of the entire small intestine, contents were flushed, and the entire small intestinal weight and length were recorded. The small intestine was then dissected into 3 segments at 10% and 85% of the total length to differentiate into the duodenum, jejunum, and ileum, respectively, and each section was weighed. Relative weights were calculated by dividing individual section weight by the total small intestinal weight per pig multiplying by 100.

### Small intestinal gene expression

On PND 35, tissue samples were collected from the mid-point of the duodenum, jejunum, and ileum, flash frozen in liquid nitrogen, and subsequently stored at -80°C pending analysis. Tissue samples were analyzed for gene expression following a similar protocol as previously described (Oelschlager et al., 2019). The TaqMan Gene Expression assay (Thermo Fisher Scientific, Waltham, MA) was utilized to perform quantitative real-time polymerase chain reaction (qRT-PCR) to quantify mRNA expression of interferon-γ (IFN-γ; NM_213948.1), tumor necrosis factor- α (TNF-α; NM_214022.1), interleukin-1β (IL-1β; NM_214055.1), zona occludens-1 (ZO-1; XM_003480423.4;), occludin (OCLN; NM_001163647.2), and claudin-1(CLDN; NM_001244539.1). Amplification by qRT-PCR was achieved for targets (IFN- γ, TNF- α, IL-1 β, ZO-1, OCLN, and CLDN) and reference (ribosomal protein L-19; RPL-19; XM_003131509.5) genes. Sample cDNA was amplified using TaqMan (4304437; Thermo Fisher Scientific, Waltham, MA) oligonucleotide probes containing 5’ fluorescent reported dye (6-FAM) and 3’ non-fluorescent quencher dye, and fluorescence was determined utilizing a QuantStudio 7 Flex Real-Time PCR System (Applied Biosystems, Foster City, CA) set to a maximum of 40 cycles. Expression of target genes was normalized through parallel amplification of endogenous threshold cycle method (Schmittgen and Livak, 2008), and results were displayed as fold change relative to the pigs in the SR-W treatment (ie, comparator group).

### Statistical analysis

Data were subjected to an analysis of variance (ANOVA) using the MIXED procedure of SAS (version 9.4; SAS institute; Cary, NC). A 2-way ANOVA was used to determine whether the overall model was significant, and in those instances, means separation was conducted assuming an alpha level of 0.05. A 3-way repeated measures ANOVA was utilized to analyze interactive and main effects of rearing environment, handling frequency, and PND on secretory IgA concentrations. Due to uneven treatment replication within each farrowing group, cohort was utilized as a random variable. Results are presented as least squares means with their respective SEM. Outliers were identified as having an absolute Studentized residual value of 3 or greater and were removed from the final dataset.

## Results

### Growth performance

Interaction and main effects for BW are displayed in **Table 3**. Throughout the entire study, there were no interaction or main effects involving handling frequency. On PND 21 and 35, there was a main effect of rearing environment with artificially-reared pigs being 1–1.5 kg heavier (*P* < 0.05) than sow-reared pigs.

**Table 3.** Effect of handling and rearing environment on pig body weights^1^

	Treatment					*P*-value		
Outcome	SR-W	SR-D	AR-W	AR-D	SEM	Handling	Rearing	Interaction
**Pigs, *n***	18	20	16	18	–	–	–	–
**Body weight, kg**								
** PND 2**	1.62	1.53	1.60	1.67	0.07	0.88	0.28	0.15
** PND 7**	2.75	2.51	2.47	2.54	0.16	0.33	0.17	0.08
** PND 14**	4.80	4.56	4.74	4.77	0.29	0.33	0.41	0.25
** PND 21**	7.21	6.96	8.47	8.51	0.51	0.69	< 0.0001	0.58
** PND 28**	9.19	9.10	10.06	9.33	0.57	0.15	0.05	0.27
** PND 35**	12.53	12.78	13.94	13.08	0.36	0.34	0.010	0.09

1 Pigs were artificially- or sow-reared, and daily- or weekly-handled from PND 2-21. On PND 21 pigs were moved to mixed-sex nursery pens containing 4–8 pigs of the same treatment. Pigs received a 5 µg/kg of BW of lipopolysaccharide on PND 28 then euthanized on PND 35 to permit sample collection. Abbreviations: AR-D, artificially reared daily handled; AR-W, artificially reared weekly handled; BW, body weight; H, handling frequency; R, rearing environment; SR-D, sow reared daily handled; SRW, sow reared weekly handled.

**Figure 2.**
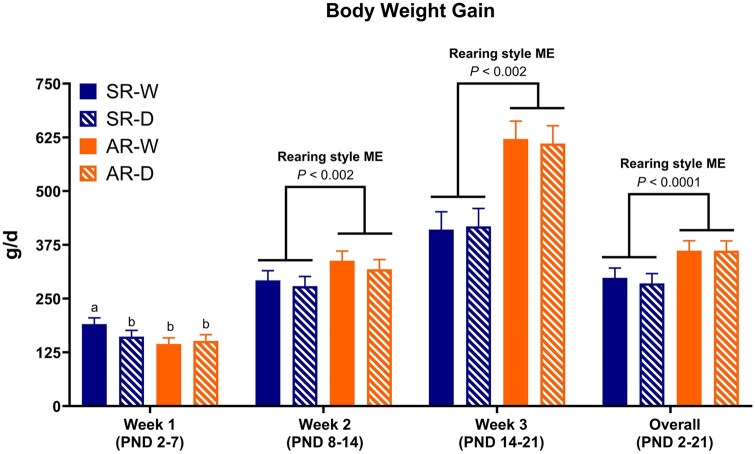
Body weight gain for the overall and individual periods during PND 2-21. Pigs were artificially- or sow-reared, and daily- or weekly-handled from PND 2-21. Abbreviations: AR-D, artificially-reared, daily-handled; AR-W, artificially-reared, weekly-handled; ME, main effect; PND, postnatal day; SR-D, sow-reared, daily-handled; SR-W, sow-reared, weekly-handled. ^a-b^Means with differing superscript letters indicate an interaction effect (*P* *<* 0.05). Main effect is denoted with bar separation, with the *P*-value displayed above.

Interaction and main effects for growth performance outcomes during PND 2-21 are displayed in **Figure 2** and **Table 4**. From PND 2-7, an interaction effect was observed as SR-W pigs had the highest (*P* < 0.05) ADG compared with all other treatments. There were no interaction effects observed during week 2 (PND 8-14), week 3 (PND 15-21), or the overall (PND 2-21) study period. However, there was a main effect of rearing environment from PND 2-7, with sow-reared pigs having increased (*P* < 0.05) ADG compared with artificially-reared pigs. From PND 8-14, 14-21, and 2-21, there were main effects of rearing environment with artificially-reared pigs having approximately 15, 49, and 24% greater (*P* < 0.05) ADG than sow-reared pigs. The main effect of handling frequency had no impact on pig growth performance during any time-point throughout this early 21-d period.

**Table 4.** Effect of handling frequency on intake and feed efficiency of artificially-reared pigs from PND 2-21^1^

	Treatment			*P*-value
Outcome	AR-W	AR-D	SEM	Handling
**Pigs, *n***	16	18	–	–
**Week 1 (PND 2-7)**				
**ADFIP g/d**	183.6	193.0	30.05	0.37
** ADFIL, g/d**	1,481	1,475	393.62	0.95
** G: F powder, g; g**	0.11	0.11	0.03	0.29
** G: F liquid, g; g**	0.81	0.82	0.12	0.78
**Week 2 (PND 8-14)**				
** ADFIP, g/d**	306.2	317.8	16.26	0.61
** ADFIL, g/d**	1,531	1,589	81.30	0.61
** G: F powder, g; g**	0.21	0.21	0.01	0.44
** G: F liquid, g; g**	1.07	1.02	0.04	0.37
**Week 3 (PND 14-21)**				
** ADFIP, g/d**	562.0	503.7	58.59	0.21
** ADFIL, g/d**	2,810	2,518	289.96	0.21
** G: F powder, g; g**	1.15	1.21	0.04	0.40
** G: F liquid, g; g**	0.23	0.24	0.01	0.41
**Overall, PND 2-21**				
** ADFIP, g/d**	362.1	347.5	25.04	0.56
** ADFIL, g/d**	1,811	1,737	125.20	0.56
** G: F powder, g: g**	0.20	0.21	0.01	0.36
** G: F liquid, g: g**	1.02	1.04	0.02	0.39

1 Pigs were artificially- or sow-reared, and daily- or weekly-handled from PND 2-21. Individual intake and feed efficiency was unavailable for sow-reared pigs, therefore only data from artificially-reared pigs are displayed. Abbreviations: ADFIL, average daily feed intake liquid; ADFIP, average daily feed intake powder; AR-D, artificially reared daily handled; AR-W, artificially reared weekly handled; G: F, gain-to-feed ratio or feed efficiency; SR-D, sow reared daily handled; SR-W, sow reared weekly handled.

Interaction and main effects for growth performance during the PND 21-35 period, after handling and rearing interventions ceased, are displayed in **Table 5**. It is important to note that during this period, the experimental unit for feed intake and G: F changed from individual pig to pen. Throughout all time-points in the PND 21-35 period, there was no interaction effect or main effect of handling frequency. From PND 21-28 there was a main effect of previous rearing environment as sow-reared pigs had increased (*P* < 0.05) ADG and higher (*P* < 0.05) G: F than artificially-reared pigs. During the overall period (PND 21-35), a main effect of previous rearing environment was observed, as sow-reared pigs had an increase (*P* < 0.05) in G: F compared with artificially-reared pigs.

**Table 5.** Effects of previous rearing environment and handling frequency on growth performance of pigs from PND 21-35^1^

	Treatment					*P*-value		
Outcome	SR-W	SR-D	AR-W	AR-D	SEM	Handling	Rearing	Interaction
**Pens, *n***	3	3	2	3	-	-	-	-
**Week 4 (PND 21-28)**								
** ADG, g/d**	289.0	308.1	150.3	222.9	45.53	0.29	0.030	0.52
** ADFI, g/d**	323.9	325.6	299.9	298.5	36.56	1.00	0.45	0.96
** G: F, g; g**	0.89	0.92	0.63	0.47	0.09	0.42	0.003	0.28
**Week 5 (PND 28-35)**								
** ADG, g/d**	433.9	502.9	581.5	458.0	88.67	0.64	0.40	0.14
** ADFI, g/d**	651.9	725.6	748.8	699.0	81.70	0.85	0.59	0.34
** G: F, g; g**	0.68	0.74	0.71	0.73	0.08	0.58	0.90	0.75
**Overall period (PND 21-35)**								
** ADG, g/d**	374.7	408.4	365.9	337.5	55.63	0.95	0.40	0.50
** ADFI, g/d**	493.0	530.7	524.3	489.4	50.95	0.98	0.91	0.44
** G: F. g: g**	0.76	0.79	0.69	0.66	0.05	0.98	0.026	0.53

1 Pigs were artificially- or sow-reared, and daily- or weekly-handled from PND 2-21. On PND 21 pigs were moved to mixed-sex nursery pens containing 4–8 pigs of the same treatment. Pigs received a 5 µg/kg of BW of lipopolysaccharide on PND 28 then euthanized on PND 35 to permit sample collection. Abbreviations: ADG, average daily body weight gain; ADFI, average daily feed intake; AR-D, artificially reared daily handled; AR-W, artificially reared weekly handled; BW, body weight; G: F, gain-to-feed ratio or feed efficiency; H, handling frequency; PND, postnatal day; R, rearing environment; SR-D, sow reared daily handled; SR-W, sow reared weekly handled.

### Fecal secretory IgA

**Figure 3.**
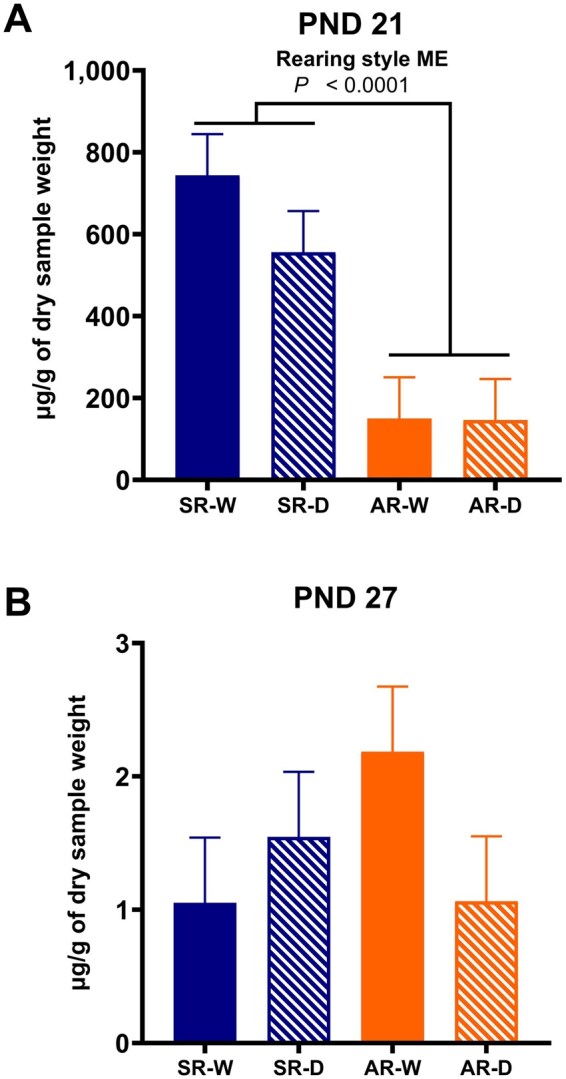
Secretory IgA concentration within the feces of pigs collected on PND 21 or 27. Pigs were artificially- or sow-reared, and daily- or weekly-handled from PND 2-21. Abbreviations: AR-D, artificially-reared, daily-handled; AR-W, artificially-reared, weekly-handled; H, handling; IgA, immunoglobulin A; ME, main effect; PND, postnatal day; R, rearing; SR-D, sow-reared, daily-handled; SR-W, sow-reared, weekly-handled. Main effect is denoted with bar separation, with the *P*-value displayed above.

Interaction and time-dependent main effects on fecal secretory IgA concentrations are displayed in **Figure 3**. No interaction or main effects involving handling frequency were observed for fecal secretory IgA on PND 21, which was prior to termination of experimental interventions. However, a main effect of rearing environment was observed with sow-reared pigs having a 502 μg/g increased (*P* < 0.05) concentration of fecal secretory IgA compared with artificially-reared pigs. Interaction and main effects were not observed on fecal secretory IgA concentrations on PND 27, which was after experimental interventions ended and pigs were transitioned to a group housing context.

**Figure 4.**
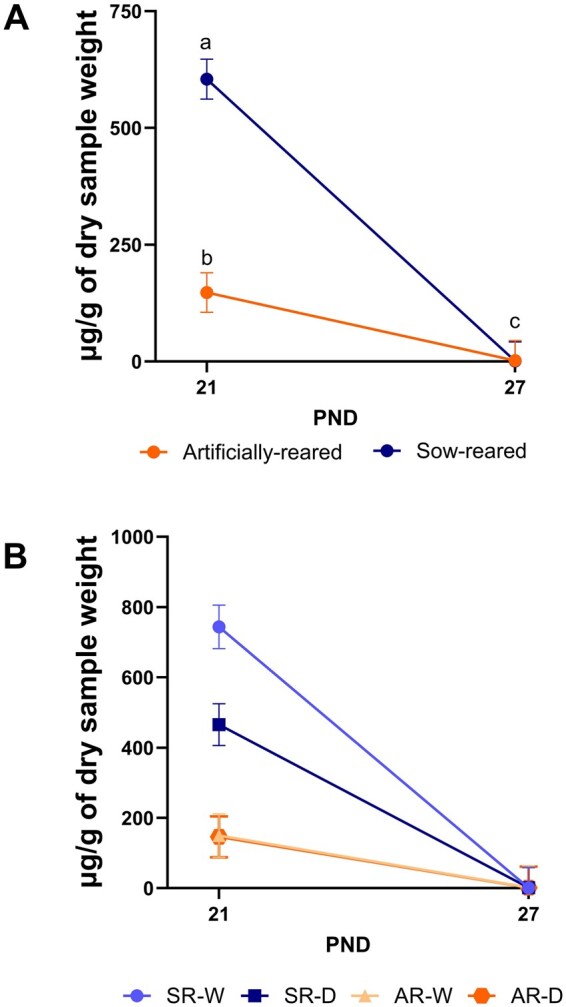
Concentration of secretory immunoglobulin A within the feces of pigs across PND 21 and 27. Pigs were artificially- or sow-reared, and daily- or weekly-handled from PND 2-21. Abbreviations: AR-D, artificially-reared, daily-handled; AR-W, artificially-reared, weekly-handled; DMB, dry matter basis; H, handling; IgA, immunoglobulin A; PND, postnatal day; R, rearing; SR-D, sow-reared, daily-handled; SR-W, sow-reared, weekly-handled. ^a–c^Means with differing superscripts indicate an interaction effect (*P* *<* 0.05).

Interaction effects for secretory IgA concentrations across time (PND 21 and 27) are displayed in **Figure 4**. No interaction effects of PND × rearing environment × handling frequency or handling frequency × PND were observed. An interaction effect of rearing environment × PND was observed, with SR pigs having the highest (*P* < 0.05) concentration of IgA on PND 21 (530.2 μg/g) compared with AR pigs PND 21 and SR and AR pigs 27 (1.3 and 2.0 μg/g, respectively). Furthermore, AR pigs had higher (*P* < 0.05) fecal IgA concentrations on PND 21 compared with SR and AR pigs on PND 27. The main effect of PND was also observed, as PND 21 had the highest (*P* < 0.05) concentration of fecal IgA at 339.1 μg/g, compared with PND 27 with a concentration of 1.7 μg/g.

### Small intestinal organ metrics

Two weeks after experimental interventions ceased, intestinal organ weights and lengths were analyzed. Interaction and main effects on organ metrics are displayed in **Table 6**. No interaction effects were observed for organ metrics. However, there was a main effect of previous rearing environment, as artificially-reared pigs had 1.6% heavier (*P* < 0.05) absolute duodenal mass and 7.3% lighter (*P* < 0.05) ileal mass compared with sow-reared pigs. Furthermore, there was a main effect of previous rearing environment with sow-reared pigs having heavier (*P* < 0.05) relative ileal weights compared with artificially-reared pigs. The main effect of previous handling frequency had no impact on either relative or absolute small intestinal weights. However, the main effect of previous handling frequency was observed for small intestinal lengths, with weekly-handled pigs having a longer (*P* < 0.05) small intestinal tract length when compared with daily-handled pigs. There was no main effect of previous rearing environment on small intestinal length.

**Table 6.** Effect of previous handling frequency and rearing environment on small intestinal metrics of pigs on PND 35^1^

	Treatment					*P*-value		
Outcome	SR-W	SR-D	AR-W	AR-D	SEM	Handling	Rearing	Interaction
**Pigs, n**	18	20	16	18	–	–	–	–
**Absolute weights**								
** Duodenum, g**	45.6	43.7	49.9	46.7	2.62	0.07	0.012	0.67
** Jejunum, g**	302.5	305.0	333.4	309.5	22.23	0.30	0.09	0.21
** Ileum, g**	86.3	85.5	82.5	77.0	3.19	0.32	0.043	0.42
** Total tract, g**	647.9	682.1	678.2	658.4	245.64	0.70	0.86	0.16
**Relative weights**								
** Duodenum, %**	0.36	0.35	0.37	0.38	0.02	0.93	0.14	0.57
** Jejunum, %**	2.38	2.33	2.48	2.42	0.13	0.51	0.23	0.92
** Ileum, %**	0.68	0.67	0.64	0.60	0.02	0.21	0.005	0.65
** Total tract, %**	5.08	5.16	4.76	5.07	1.73	0.30	0.27	0.56
**Absolute length**								
** Total tract, cm**	1,325	1,249	1,335	1,282	25.09	0.009	0.37	0.63

1 Pigs were artificially- or sow-reared, and daily- or weekly-handled from PND 2-21. On PND 21 pigs were moved to mixed-sex nursery pens containing 4–8 pigs of the same treatment. Pigs received a 5 µg/kg of BW of lipopolysaccharide on PND 28 then euthanized on PND 35 to permit sample collection. Abbreviations: AR-D, artificially reared daily handled; AR-W, artificially reared weekly handled; H, handling frequency; PND, postnatal day; R, rearing environment; SR-D, sow reared daily handled; SR-W, sow reared weekly handled.

### Intestinal gene expression of cytokines and tight junction proteins

Gene (ie, mRNA) expression of cytokine and tight-junction proteins were evaluated two weeks after rearing environment and handling frequency interventions ended, and after the transition to a novel environment, dry diet, and mixing with novel conspecifics. Interaction and main effects on gene expression of cytokines and tight junction proteins are displayed in **Table 7**. No interaction effects were observed in any small intestinal sections for expression of either cytokine or tight junction proteins. However, near significance was observed as AR-D pigs displayed higher (*P* = 0.05) expression of TNF-α in duodenal tissue compared with all other treatments. Within the jejunum, TNF-α expression was decreased (*P* = 0.05) in AR-W pigs when compared with SR-W pigs, while daily-handled groups (ie, AR-D and SR-D pigs) were intermediary.

**Table 7.** Effects of previous rearing environment and handling frequency on gene expression in small intestinal tissue of pigs on PND 34^1^

	Treatment					*P*-value		
Outcome	SR-W	SR-D	AR-W	AR-D	SEM	Handling	Rearing	Interaction
**Duodenum**								
** IFN-γ**	1.00	1.73	1.33	1.21	0.21	0.40	0.99	0.22
** TNF-α**	1.00	1.14	0.83	2.02	0.28	0.018	0.18	0.05
** IL1-β**	1.00	1.03	0.57	0.94	0.23	0.23	0.37	0.28
** ZO-1**	1.00	2.09	0.94	1.97	0.43	0.029	0.86	0.96
** OCLD**	1.00	1.13	0.69	1.60	0.24	0.028	0.73	0.09
** CLDN**	1.00	1.30	0.74	0.82	0.25	0.44	0.12	0.63
**Jejunum**								
** IFN-γ**	1.00	0.19	1.23	0.57	0.41	0.06	0.43	0.85
** TNF-α**	1.00	0.68	0.46	0.79	0.18	0.96	0.18	0.05
** IL1-β**	1.00	0.78	1.45	1.10	0.31	0.12	0.41	0.43
** ZO-1**	1.00	0.97	1.20	0.56	0.30	0.23	0.72	0.28
** OCLD**	1.00	0.84	0.57	1.00	0.17	0.41	0.42	0.09
** CLDN**	1.00	1.07	1.38	1.18	0.41	0.86	0.53	0.72
**Ileum**								
** IFN-γ**	1.00	0.58	1.92	1.35	0.44	0.19	0.032	0.92
** TNF-α**	1.00	0.98	1.24	1.08	0.22	0.67	0.42	0.71
** IL1-β**	1.00	1.18	2.25	1.40	0.31	0.22	0.021	0.11
** ZO-1**	1.00	0.73	0.76	0.96	0.14	0.82	0.98	0.09
** OCLD**	1.00	0.88	1.07	1.32	0.25	0.78	0.29	0.45
** CLDN**	1.00	1.11	0.76	0.45	0.31	0.75	0.13	0.48

1 Pigs were artificially- or sow-reared, and daily- or weekly-handled from PND 2-21. On PND 21 pigs were moved to mixed-sex nursery pens containing 4–8 pigs of the same treatment. Pigs received a 5 µg/kg of BW of lipopolysaccharide on PND 28 then euthanized on PND 35 to permit sample collection. Abbreviations: AR-D, artificially reared daily handled; AR-W, artificially reared weekly handled; CLDN, claudin-1; H, handling frequency; IFN, interferon; IL, interleukin; OCLD, occludin; PND, postnatal day; R, rearing environment; SR-D, sow-reared daily handled; SR-W, sow reared handled weekly; TNF, tumor necrosis factor; ZO, zonula occludens.

A main effect of previous handling frequency was observed in the duodenum with daily-handled pigs having higher (*P* < 0.05) expression of TNF- α, ZO-1, and OCLD than weekly-handled pigs. There was no impact of previous rearing environment on gene expression within the duodenum or jejunum. Furthermore, there was no impact of previous handling frequency on jejunal or ileal gene expression. The main effect of previous rearing environment was observed in the ileum, with artificially-reared pigs displaying higher (*P* < 0.05) expression of IFN-γ, and IL1-β when compared with sow-reared pigs.

### Clinical chemistry and hematology parameters

Interaction effects on hematological and clinical chemistry outcomes on PND 14, while pigs differed in rearing environment and handling frequency, are displayed in **Table 8**. An interaction effect was observed for red blood cell counts, with SR-W pigs having a higher (*P* < 0.05) count compared with AR-D pigs, while SR-D pigs were intermediate, and AR-W having the lowest (*P* < 0.05) count compared with all other treatments. SR-W and SR-D pigs had increased (*P* < 0.05) mean corpuscular hemoglobin concentration levels when compared with both AR-W and AR-D, while AR-D had the lowest (*P* < 0.05) level. The percentage of basophils decreased (*P* < 0.05) in the AR-D and SR-D pigs compared with AR-W pigs, while SR-W pigs were intermediate. Total protein and globulin levels were increased (*P* < 0.05) in AR-D pigs compared with all other treatments. SR-D pigs had the highest (*P* < 0.05) total bilirubin concentration when compared with all other treatments, while this outcome was increased (*P* < 0.05) in SR-W pigs compared with AR-D and AR-W pigs.

**Table 8.** Effect of rearing environment and handling frequency on hematological outcomes of pigs on PND 14^1^

	Treatment					*P*-value		
Outcome	SR-W	SR-D	AR-W	AR-D	SEM	Handling	Rearing	Interaction
**Pigs, *n***	18	20	16	18	–	–	–	–
**Total and differential cell counts**								
** RBC count, ×10^6^ cells/µL**	5.7^a^	5.5^ab^	4.8^c^	5.3^b^	0.13	0.19	<0.0001	0.005
** Hemoglobin, g/dL**	11.2	11.1	9.2	9.6	0.23	0.37	<0.0001	0.24
** Hematocrit**	34.8	34.7	29.9	32.0	1.31	0.17	<0.0001	0.10
** MCV, fl**	61.5	63.4	63.6	65.1	1.89	0.11	0.07	0.83
** MCH, pg**	19.6	20.4	19.0	18.7	0.47	0.52	0.003	0.11
** MCHC, g/dL**	31.7^a^	32.2^a^	29.9^b^	29.0^c^	0.40	0.35	<0.0001	0.007
** Platelets, ×10^3^ platelets/µL**	684.8	617.5	645.6	675.8	66.03	0.64	0.81	0.23
** MPV**	10.6	10.8	10.3	10.8	0.37	0.18	0.50	0.54
** WBC count, ×10^3^ cells/µL**	8.6	8.9	7.7	8.8	0.77	0.28	0.41	0.52
** Segmented neutrophils, % **	36.8	37.3	42.3	41.8	4.15	1.00	0.10	0.88
** Lymphocytes, %**	57.9	57.6	51.5	52.3	3.77	0.93	0.08	0.86
** Monocytes, %**	3.3	2.5	4.4	5.2	1.19	0.96	0.010	0.28
** Eosinophils, % **	1.9	1.0	0.7	0.3	0.55	0.07	0.004	0.43
** Basophils, %**	0.25^ab^	0.21^b^	0.57^a^	0.00^b^	0.15	0.014	0.65	0.037
**Clinical Chemistry**								
** Creatine, mg/mL**	0.72	0.72	0.65	0.66	0.03	0.95	0.043	0.88
** BUN, mg/dL**	6.1	5.6	10.2	9.7	0.58	0.33	<0.0001	1.00
** Total protein, g/dL**	4.5^b^	4.4^b^	4.5^b^	5.00^a^	0.13	0.14	0.017	0.008
** Albumin, g/dL**	2.8	2.8	2.9	3.0	0.11	0.99	0.09	0.34
** Globulin, g/dL**	1.7^b^	1.6^b^	1.6^b^	2.0^a^	0.14	0.07	0.07	0.002
** Albumin: globulin ratio**	1.8	1.8	1.8	1.6	0.16	0.36	0.29	0.36
** Calcium, mg/dL**	11.3	11.1	11.6	12.1	0.22	0.36	0.003	0.07
** Phosphorus, mg/dL**	10.5	10.6	10.2	9.4	0.42	0.11	0.0008	0.06
** Sodium, mmol/L**	137.4	137.6	144.5	145.2	1.20	0.58	<0.0001	0.75
** Potassium, mmol/L**	5.8	6.2	7.7	7.5	0.23	0.69	<0.0001	0.07
** Sodium: potassium ratio**	23.8	22.6	19.2	20.1	0.94	0.79	<0.0001	0.09
** Chloride, mmol/L**	102.9	102.7	106.0	105.5	0.86	0.52	<0.0001	0.84
** Glucose, mg/dL**	133.2	128.0	138.4	134.7	4.95	0.17	0.07	0.81
** AST, U/L**	40.5	52.8	35.5	37.2	3.93	0.024	0.002	0.31
** GGT, U/L**	23.2	33.6	24.2	25.8	7.21	0.022	0.19	0.110
** Total bilirubin, mg/dL**	0.40^b^	0.50^a^	0.19^c^	0.17^c^	0.03	0.11	<0.0001	0.028
** CPK, U/L**	459.7	696.2	371.3	483.4	143.52	0.08	0.12	0.53
** Cholesterol total, mg/dL**	185.4	176.0	90.3	93.4	11.15	0.77	<0.0001	0.55
** Magnesium, mg/dL**	2.5	2.6	2.8	2.9	0.07	0.16	0.0003	0.0003
** Triglycerides, mg/dL**	108.2	108.9	90.0	77.1	7.76	0.40	0.001	0.36

1 Pigs were artificially- or sow-reared, and daily- or weekly-handled from PND 2-21. On PND 21 pigs were moved to mixed-sex nursery pens containing 4–8 pigs of the same treatment. Pigs received a 5 µg/kg of BW of lipopolysaccharide on PND 28 then euthanized on PND 35 to permit sample collection. Abbreviations: APT, alkali denaturation test; AR-D, artificially reared handled daily; AR-W, artificially reared handled weekly; AST, aspartate aminotransferase; BUN, blood urea nitrogen; CPK, creatine phosphokinase; GGT, gamma-glutamyl transferase; H, handling frequency; MCH, mean corpuscular hemoglobin; MCHC, mean corpuscular hemoglobin concentration; MCV, mean corpuscular volume; MPV, mean platelet volume; PND, postnatal day; R, rearing environment; RBC, red blood cells; SR-D, sow reared handled daily; SR-W, sow reared handled weekly; WBC, white blood cells.

a–c Means lacking a common superscript letter differ (*P *< 0.05).

Main effects on hematological and clinical chemistry outcomes on PND 14 are also displayed in **Table 8**. There was a main effect of handling frequency on the percentage of basophils present with weekly-handled pigs having more (*P* < 0.05) than the daily-handled pigs. However, daily-handled pigs had higher (*P* < 0.05) levels of aspartate aminotransferase and gamma-glutamyl transferase when compared with weekly-handled pigs. A main effect of rearing environment was observed with sow-reared pigs having increased (*P* < 0.05) levels of red blood cells, hemoglobin, hematocrit, mean corpuscular hemoglobin, mean corpuscular hemoglobin concentration, and percentage of eosinophils when compared with artificially-reared pigs. However, artificially-reared pigs had increased (*P* < 0.05) percentage of monocytes when compared with the sow-reared pigs. Artificially-reared pigs had increased (*P* < 0.05) blood urea nitrogen compared with sow-reared pigs.

Interaction effects on hematological and clinical chemistry parameters on PND 35, two weeks after early-life interventions ceased, are displayed in **Table 9**. An interaction effect was observed where SR-D pigs had the highest (*P* < 0.05) concentration of mean corpuscular volume compared with SR-W, while AR-D and AR-W were intermediate. SR-W pigs had the lowest (*P* < 0.05) level of mean corpuscular hemoglobin compared with all other treatments, while the percentage of eosinophils was highest (*P* < 0.05) in the AR-W pigs compared with all other treatments. AR-W pigs had the lowest (*P* < 0.05) potassium level compared with all other treatments. However, AR-W pigs had the highest sodium-to-potassium ratio when compared with SR-W and AR-D pigs, with SR-D pigs being intermediate.

**Table 9.** Effect of previous rearing environment and handling frequency on hematological outcomes of pigs on PND 35^1^

	Treatment					*P*-value		
Outcome	SR-W	SR-D	AR-W	AR-D	SEM	Handling	Rearing	Interaction
**Pigs, *n***	18	20	16	18	–	–	–	–
**Total and differential cell counts**								
** RBC count, ×10^6^ cells/µL**	6.0	5.5	5.4	5.2	0.14	0.002	0.0004	0.28
** Hemoglobin, g/dL**	10.0	9.9	9.9	9.4	0.25	0.16	0.13	0.37
** Hematocrit**	31.9	31.0	30.1	28.1	0.64	0.021	0.0002	0.35
** MCV, fl**	53.9^b^	56.7^a^	55.5^ab^	55.1^ab^	1.42	0.09	0.98	0.030
** MCH, pg**	17.0^b^	18.1^a^	18.2^a^	18.1^a^	0.67	0.049	0.011	0.020
** Platelets, ×10^3^ platelets/µL**	607.1	608.9	546.2	618.1	30.68	0.21	0.38	0.23
** MPV**	10.1	10.4	10.8	10.6	0.28	0.79	0.026	0.16
** Segmented neutrophils, % **	52.4	47.8	52.8	57.3	3.07	0.98	0.08	0.10
** Lymphocytes, %**	43.6	48.8	41.5	38.3	2.68	0.67	0.016	0.10
** Monocytes, %**	4.9	3.9	6.3	5.2	1.50	0.12	0.06	0.93
** Eosinophils, % **	0.01^b^	0.02^b^	0.37^a^	0.02^b^	0.09	0.014	0.011	0.008
** Basophils, %**	0.14	0.13	0.24	0.19	0.13	0.77	0.41	0.89
**Clinical Chemistry**								
** Creatine, mg/mL**	0.65	0.66	0.59	0.60	0.04	0.73	0.002	0.93
** BUN, mg/dL**	6.6	5.4	6.2	6.4	1.17	0.43	0.40	0.19
** Total protein, g/dL**	4.2	4.1	4.3	1.4	0.07	0.83	0.003	0.20
** Albumin, g/dL**	3.0	3.0	3.3	3.3	0.06	0.36	<0.0001	0.69
** Globulin, g/dL**	1.1	1.1	1.0	1.0	0.05	0.23	0.0003	0.39
** Albumin: globulin ratio**	2.7	2.6	3.4	3.1	0.13	0.06	<0.0001	0.33
** Calcium, mg/dL**	10.5	10.4	10.9	10.5	0.28	0.11	0.13	0.18
** Phosphorus, mg/dL**	9.2	9.3	8.4	9.4	0.38	0.06	0.16	0.13
** Sodium, mmol/L**	141.0	141.5	140.8	140.2	1.10	0.96	0.038	0.11
** Potassium, mmol/L**	5.0^a^	4.7^a^	4.4^b^	5.0^a^	0.13	0.13	0.18	0.0003
** Sodium: potassium ratio**	28.7^bc^	30.6^ab^	32.3^a^	28.2^c^	0.75	0.12	0.38	<0.001
** Chloride, mmol/L**	102.9	102.9	101.9	101.8	1.01	0.77	0.003	0.91
** Glucose, mg/dL**	126.3	120.6	141.1	118.8	17.3	0.017	0.26	0.16
** AST, U/L**	33.0	33.7	27.1	26.6	4.92	0.97	0.005	0.80
** GGT, U/L**	32.0	37.8	26.1	35.6	10.32	0.034	0.26	0.61
** Total bilirubin, mg/dL**	0.36	0.52	0.18	0.59	0.21	0.0006	0.50	0.13
** CPK, U/L**	925.5	996.7	719.5	623.2	98.02	0.89	0.002	0.36
** Cholesterol total, mg/dL**	62.5	69.7	60.4	70.5	5.35	0.0005	0.79	0.54
** Magnesium, mg/dL**	2.2	2.3	2.1	2.3	0.14	0.029	0.05	0.15
** Triglycerides, mg/dL**	26.7	26.0	16.9	23.7	9.92	0.24	0.023	0.16

1 Pigs were artificially- or sow-reared, and daily- or weekly-handled from PND 2-21. On PND 21 pigs were moved to mixed-sex nursery pens containing 4–8 pigs of the same treatment. Pigs received a 5 µg/kg of BW of lipopolysaccharide on PND 28 then euthanized on PND 35 to permit sample collection. Abbreviations: APT, alkali denaturation test; AR-D, artificially reared daily handled; AR-W, artificially reared weekly handled; AST, aspartate aminotransferase; BUN, blood urea nitrogen; CPK, creatine phosphokinase; GGT, gamma-glutamyl transferase; H, handling frequency; MCH, mean corpuscular hemoglobin; MCHC, mean corpuscular hemoglobin concentration; MCV, mean corpuscular volume; MPV, mean platelet volume; R, rearing environment; RBC, red blood cells; SR-D, sow reared daily handled; SR-W, sow reared weekly handled.

a–c Means lacking a common superscript letter differ (*P *< 0.05).

Main effects of experimental treatment on hematological and clinical chemistry parameters of 35-d-old pigs are also displayed in **Table 9**. There was a main effect of previous handling frequency observed as weekly-handled pigs had increased (*P* < 0.05) red blood cell count, hematocrit, percentage of eosinophils, and glucose concentrations compared with daily-handled pigs. Pigs that were previously handled daily, however, had increased (*P* < 0.05) mean corpuscular hemoglobin, gamma-glutamyl transferase, total bilirubin, total cholesterol, and magnesium compared with weekly-handled pigs. The main effect of rearing environment was observed with previously sow-reared pigs exhibiting increased (*P* < 0.05) hematocrit, red blood cell counts, and percentage of lymphocytes when compared previously artificially-reared pigs. Artificially-reared pigs had increased (*P* < 0.05) mean corpuscular hemoglobin concentration, mean platelet volume, and percentage of eosinophils compared with sow-reared pigs.

In terms of clinical chemistry parameters, a main effect of previous rearing environment was observed. Artificially-reared pigs had increased (*P* < 0.05) levels of albumin and albumin-to-globulin ratio compared with sow-reared pigs. However, sow-reared pigs exhibited an increased (*P* < 0.05) globulin concentration when compared with artificially-reared pigs. Sow-reared pigs had increased (*P* < 0.05) levels of creatine and creatine phosphate kinase when compared with artificially-reared pigs. Sow-reared pigs also exhibited increased (*P* < 0.05) concentrations of total protein, triglycerides, sodium, and chloride relative to artificially-reared pigs. Liver enzymes, including aspartate aminotransferase, were increased (*P* < 0.05) in sow-reared pigs compared with artificially-reared pigs.

## Discussion

Improvements in genetic selection and farm management in recent years have resulted in increased sow litter sizes, where the number of pigs born alive may exceed the number of teats available for suckling (Baxter et al., 2013; Zhang et al., 2021). An inability to claim a teat can result in insufficient intake leading to undernutrition, poor growth, hypothermia, morbidity, and potentially, mortality in young pigs (Le Dividich et al., 2005; Zhang et al., 2021). To reduce pre-weaning mortality due to insufficient intake, cross-fostering or artificial rearing may be implemented. Artificial rearing is the process of removing the pigs from the sow and placing them in a separate area, then providing all necessities for growth and health in the absence of the dam. This separate rearing environment often differs significantly from the farrowing crate in terms of temperature, flooring, lighting, and social dynamics, which can contribute to the overall stress response and developmental outcomes in young pigs. However, artificially-rearing pigs may elicit a variety of factors that can activate stress mechanisms through early removal from the sow, increased human interaction, and exposure to a novel environment (Schmitt et al., 2019a). However, there is potential for such exposure to early-life stress to increase stress resiliency in contexts where the same mechanisms are activated again, such as when pigs are moved into nursery pens, mixed with novel conspecifics, and transitioned to a dry diet. Therefore, we hypothesized that pigs that were artificially reared and handled daily would exhibit greater body weights throughout the study, enhanced growth performance, and adapt faster to stress brought on by the transition to group housing, dry diets, and mixing with novel conspecifics due to increased resiliency compared with sow-reared and weekly-handled pigs.

### Growth performance

Contrary to our hypothesis, the artificially-reared and daily-handled context did not elicit clear advantages over other interventions in terms of final body weights or growth performance. However, early-life rearing environment influenced early weight gain, with artificially-reared pigs exhibiting reduced growth during the first week of the study, likely due to the stress associated with transitioning to the artificial rearing facility and removal from the sow. Exposure to psychological stress within 2 d after birth may have resulted in the activation of the hypothalamic-pituitary-adrenal axis, leading to the release of glucocorticoids. Although the hypothalamic-pituitary-adrenal axis may not be fully developed until after 35 days of age (Kanitz et al., 2003), pigs are still capable of mounting a glucocorticoid response to stress, as observed prenatally (Klemcke, 1995) and during the first week of life (Kanitz et al., 1999; Kanitz et al., 2003). Increased levels of glucocorticoids present within the blood can act upon the endocrine system due to the bidirectional communication of the gut-brain axis, altering metabolic processes within the pig. Elevated glucocorticoids have been reported to increase the expression of leptin (Meadus et al., 2002; Ramsay and Richards, 2004), thereby suppressing appetite signals, which may explain the decrease in body weight gain during the first week for artificially-reared pigs. However, in our study, metabolic hormones and intake between different rearing environments were not measured to validate this hypothesis.

After 5 days in the facility, artificially-reared pigs demonstrated increased weight gain through PND 21. This enhanced growth advantage resulted in significantly heavier body weights during the nursery phase, with artificially-reared pigs weighing 1.5, 0.5, and 1.0 kg more than sow-reared pigs on PND 21, 28, and 35, respectively. However, the difference in body weight between treatments may have been greater had competition for teat access among sow-reared pigs not been mitigated by the removal of litter mates. In the current study, pigs from each litter were allocated to both rearing environments, resulting in each sows’ number of teats exceeding the number of nursing pigs. Thus, reducing sow-reared pigs competition for teats and increasing their ability to select an anterior teat which typically yields more milk than posterior teats (Gill and Thomson, 1956; Fraser, 1984; Kim et al., 2000; Blavi et al., 2021; Amdi et al., 2022). Notably, the increased body weight on PND 21 helped maintain artificially-reared pigs advantage throughout the nursery phase, highlighting the substantial impact of the rearing environment on longitudinal growth trajectories.

These results are in contrast with previous research from our lab, which reported no significant differences in body weight during either the suckling or nursery phase as a result of rearing environment (Fil et al., 2021). However, our results align with observations by Vergauwen et al. (2017), who reported that artificially-reared pigs were heavier at PND 19 compared with sow-reared pigs. The growth rate of sow-reared pigs may be constrained by factors such as the timing and volume of milk intake and the influence of teat position on the sow. Sows release milk approximately every 40 minutes, with each let-down lasting a maximum of 20 seconds (Fraser, 1980), providing only brief opportunities for piglets to consume sufficient milk for growth and maintenance. Additionally, anterior teats produce more milk than posterior teats (Barber et al., 1955; Gill & Thomson, 1956), further contributing to growth disparities among piglets. In contrast, artificially-reared pigs were able to dictate both the timing and volume of milk consumption, thereby removing the sow as a potential limiting factor for growth. This corresponds to Sommer et al. (2025), who reported that pigs fed either ad libitum or according to body weight to mimic milk intake on the sow (ie, ‘prescribed’ feeding) consumed a similar volume of milk when normalized to body weight; however, body weight–based feeding resulted in slower growth.

Although artificially-reared pigs maintained a higher body weight throughout the study, they experienced a decline in body weight gain and feed efficiency after transitioning to a dry diet and group housing in the nursery phase (PND 21-28). This decline was likely due to a combination of dietary stress, caused by the introduction of dry diets, and psychological stress from exposure to an unfamiliar environment and novel conspecifics. The shift from a highly digestible milk or milk replacer to a dry diet is known to alter intestinal morphology, and induce intestinal stress (Moeser et al., 2007) and dysbiosis in young pigs (Greese et al., 2017). Furthermore, psychological stressors, such as mixing with unfamiliar conspecifics, can contribute to dysbiosis by triggering increased agonistic behavior and elevated cortisol levels (de Groot et al., 2021; Merlot et al., 2004; Li et al., 2017), which impairs weight gain and feed efficiency (Mei et al., 2016; Mcglone and Curtis, 1985; Li et al., 2017).

Activation of the hypothalamic-pituitary-adrenal axis in response to stress can result in homeostatic dysregulation within the gut, disrupting immune function and impairing nutrient digestion and absorption (Frese et al., 2015; Nowland et al., 2022; Qi et al., 2020). The microbiota-gut-brain axis regulates key processes including energy balance, intestinal integrity, immunity, and neurodevelopment (Barko et al., 2018; Guevarra et al., 2019, Joung et al., 2024). Furthermore, dysbiosis may permit pathogen proliferation, leading to disorders like post-weaning diarrhea or inflammatory bowel disease (Li et al., 2017; Wang et al., 2019). This microbial shift and immune activation may explain reduced weight gain in the artificially-reared pigs, despite similar feed intake as the sow-reared pigs as energy may be redirected toward immune responses (Greese et al., 2017). While these effects may be transient, the long-term impact on health and performance remains uncertain, warranting further research.

### Fecal secretory IgA

Secretory IgA is a key immunoglobulin associated with the intestinal mucosa, where it serves as a primary defense mechanism against pathogenic and infectious agents (Lamm, 1976; Snoeck et al., 2006). Consequently, elevated levels of IgA in feces or serum can offer valuable insight into intestinal health and enteric disruptions. In the present study, sow-reared pigs exhibited the highest fecal secretory IgA concentrations on PND 21. This aligns with the work performed by Han et al. (2024), who reported that sow-reared pigs raised through PND 35 had higher serum IgA compared with artificially-reared pigs reared starting on PND 7. This increase in IgA likely resulted from greater exposure to pathogens through contact with the sow and littermates, as well as the passive transfer of IgA via colostrum and milk. At this developmental stage, pigs have an immature immune system (Lamm, 1976), and in parallel, IgA is the predominant milk immunoglobulin that helps to train offspring immunity regarding environmental antigens (Bourne and Curtis, 1973). In contrast to sow-reared pigs, artificially-reared pigs were housed individually in our research context, which limits their exposure to pathogens due to the absence of direct contact with other pigs, minimal interaction with caretakers, and separation from most waste products due to the use of vinyl-coated, expanded metal flooring in the home-cage environment. Concurrently, artificially-reared pigs consumed milk replacer that lacked porcine maternal IgA and other immunoglobulins, effectively negating exogenous intake of IgA. As a result, the consumption of milk from the sow and exposure to novel pathogens likely contributed to the increased secretory IgA levels observed in the feces of sow-reared pigs, a mechanism also observed in the serum of pigs consuming milk (Cao et al., 2022). Furthermore, there were no differences in fecal secretory IgA concentrations on PND 27, with the implication that increased the IgA concentration on PND 21 was likely a transient response to the early-life exposure to environmental pathogens and maternal milk in sow-reared pigs.

### Small intestinal organ metrics

The small intestine plays a crucial role in nutrient digestion and absorption, immune cell secretion, and barrier protection. It consists of various cell types that influence intestinal structure and function, including nutrient digestion/absorption and immune response as integrated with the microbiota-gut-brain axis (Latorre et al., 2016). Increases in intestinal length or maturation may enhance nutrient absorption by expanding overall absorptive surface area, with implications toward both growth and immunity. Within the small intestine, the ileum is particularly important for completing nutrient and bile salt absorption (Latorre et al., 2016).

Previous literature offers insights into the effects of rearing environment on organ metrics, though a gap in understanding exists on the influence of animal handling. To our knowledge, no previous studies have explored the impact of handling frequency on intestinal metrics, such as small intestinal tract length. In the present study, small intestinal metrics were minimally affected by treatment, with small intestinal length varying by handling frequency and relative ileal weights differing based on rearing environment. The observed influence of rearing environment in our study contrasts with research from de Greeff et al. (2016) and De Vos et al. (2014), where no significant differences were reported in relative intestinal weight between artificially-reared and sow-reared pigs. Although sow-reared pigs exhibited greater relative ileal weights, these differences may not hold biological significance, as both values were less than 1% of total body weight, differing by only 0.06 percentage units. However, the increased ileal weight observed in sow-reared pigs may reflect enhanced mucosal growth, enzyme production, or immune cell presence, which could translate to advantages in growth and/or health outcomes. Given that organ metric alterations may be linked to immune responses in pigs, we further examined the expression of tight junction proteins and cytokines across all three sections of the small intestine.

### Small intestinal gene expression

The transition to group nursery pens, a dry diet, and mixing with unfamiliar conspecifics can induce intestinal distress by reducing tight junction proteins, which compromises the integrity of the small intestinal epithelial barrier. This increase in permeability may allow the translocation of microbial components such as lipopolysaccharide, which is an endotoxin originating from the outer membrane of Gram-negative bacteria. Lipopolysaccharide can further compromise the integrity of the intestinal barrier and elicit the expression of pro-inflammatory cytokines (Cao et al., 2018). Tight junction proteins, such as claudins, zonula occludens-1, and occludin, are essential for maintaining barrier function and regulating small intestinal permeability. A reduction in these proteins increases permeability, thereby potentially allowing pathogens (e.g., bacteria, viruses, and protozoa), toxins, and antigens to pass through the barrier (Campbell et al., 2013; Wang and Ji, 2019). A breach in barrier function may concomitantly trigger inflammation through the release of pro-inflammatory cytokines (Moeser et al., 2007), which play a crucial role in subsequent recruitment of immune cells for pathogen clearance. Consequently, a decline in tight junction protein expression not only disrupts intestinal integrity but also increases susceptibility to infection and inflammation (Wang and Ji, 2019).

In the present study, gene expression of tight junction proteins in the duodenum and ileum was influenced by handling frequency and rearing environment, respectively. The limited data on handling and gene expression in pigs highlights the significance of this study in understanding enteric stress responses. In the duodenum, the expression of tight junction proteins and pro-inflammatory cytokines was elevated in daily-handled pigs, potentially indicating a more robust response to environmental, psychological, and immunological stressors to which pigs were exposed in this experimental context. Additionally, exposure to LPS after the transition to nursery pens may have contributed to the prolonged expression of cytokines as it mimics a bacterial infection, which may have contributed to an adaptive response over time. Pigs subjected to daily handling presumably experienced both psychological and immunological stressors more frequently due to routine human interactions, which may have contributed to adaptive stress responses over time. Repeated exposure to stress can initially heighten immune activation but may promote resiliency if pigs were to adapt to frequent handling (Gimsa et al., 2018).

Early-life rearing environment also influenced pro-inflammatory cytokine expression at the small intestinal tissue level, with artificially-reared pigs exhibiting higher local cytokine expression than sow-reared pigs. This aligns with results reported by Han et al. (2022), who observed increased cytokine expression in artificially-reared pigs on PND 7 or 21 compared with their sow-reared counterparts, following the exposure to novel conspecifics, a dry diet, and group housing starting on PND 35. Concomitantly, immune stimulation through lipopolysaccharide administration may have amplified the expression of cytokines. This exaggerated response in the artificially-reared pigs could reflect a lack of early microbial and immune stimulation, such as limited environmental exposure to LPS, resulting in a more reactive immune response. The sustained elevation of pro-inflammatory cytokines in the current study also supports previous research where increased cytokine expression was observed two weeks after weaning (de Greeff et al., 2016). This heightened immune response likely reflects heightened intestinal distress in artificially-reared pigs, and is likely related to our observation of reduced body weight gain during the week following transition to the group housing context.

Stress-induced dysbiosis can also trigger systemic and localized immune activation (Mu et al., 2015; Patil et al., 2020). As previously noted, transitioning to a dry diet can disrupt microbial balance, promoting pathogenic bacterial growth and immune activation via the microbiota-gut-brain axis. This multi-directional communication axis plays a critical role in stress and immune regulation in young pigs (Barko et al., 2018; Farzi et al., 2018; Li et al., 2018; Guevarra et al., 2019). As such, intestinal inflammation and dysbiosis can signal the brain to initiate neuroendocrine responses, including cortisol release. In our study, elevated IL-1β expression supports activation of this pathway, as IL-1β binding to hypothalamic receptors can stimulate corticotropin-releasing factor secretion, leading to systemic cortisol release, which can subsequently suppress the expression of pro-inflammatory cytokines (Rook, 1999; Padgett and Glaser, 2003; Pavlov et al., 2010). This neuroendocrine response is vital in the regulation of the immune system because it acts as a feedback mechanism to help the body return to homeostasis.

### Clinical chemistry and hematology parameters

Blood biomarkers provide valuable systemic insights into the health and physiological state of pigs, thereby enabling the assessment of organ and immune function, as well as early detection of physiological, metabolic, and immune disruptions (Buzzard et al., 2013; Perri et al., 2017). The innate immune system plays a key role in the swift and active defense against pathogens by recognizing pathogen associated molecular patterns (Dempsey et al., 2003; Marshall et al., 2018). Upon pathogen recognition, the innate immune system activates a variety of cells, such as basophils, eosinophils, neutrophils, and monocytes, to initiate a defense response (Dempsey et al., 2003; Turvey and Broide, 2010; Marshall et al., 2018; Pluske et al., 2018b). Basophils and eosinophils produce inflammatory cytokines, which are crucial for triggering immune cell recruitment, inflammation, and fever (Dempsey et al., 2003; Marshall et al., 2018). On PND 14, before transitioning to a novel environment, dry diet, and social interactions with conspecifics, artificially-reared pigs had the highest concentrations of circulating monocytes and basophils, perhaps indicating heightened immune responsivity. Additionally, these pigs exhibited lower eosinophil concentrations, suggesting a reduced potential for allergic reactions (Turvey and Broide, 2010; Marshall et al., 2018;).

Two weeks later, on PND 35, following exposure to novel conspecifics, environments, and diets, artificially-reared pigs that had been handled weekly exhibited elevated plasma eosinophil concentrations. Eosinophils are produced in the bone marrow and migrate through the blood to tissue in response to stimuli such as allergic reactions, chronic inflammation, and parasitic infections as observed in preclinical and clinical research (Hubscher, 1975; Jones, 1993; Zheng et al., 2009). The increase in eosinophils may reflect an altered or heightened allergic-type immune response to environmental antigens encountered in the group-housing context. This response is likely driven by the pigs’ limited early-life antigen exposure, as they were housed individually and handled infrequently. A reduced immune stimulation during early-life development may have resulted in an under-stimulated immune system, leading to an amplified response upon later exposure to novel antigens.

While there were some changes in hematological parameters (e.g., red blood cell counts, hemoglobin, mean corpuscular hemoglobin, and mean corpuscular hemoglobin concentrations) on both PND 14 and 35, none of the pigs presented with clinically-relevant outcomes. Prior to the study, each pig received a 200 mg iron dextran injection, and those that were artificially reared consumed a milk replacer with sufficient iron levels to meet physiological requirements of the young pig. Despite undergoing early-life stress, which can influence physiological and immunological responses, pigs in our study generally remained healthy, as evidenced by their blood parameters falling within established clinical reference ranges for pigs aged 7 to 35 days (Egeli et al., 1998; Perri et al., 2017; Ventrella et al., 2017). The alignment of clinical results with established reference ranges provides valuable insight into the pigs’ local and systemic responses to stress. While these pigs demonstrated local intestinal immune responses, no systemic responses were evident. By aligning clinical outcomes with previously established reference ranges, our study adds to the understanding of how stressors can shape immune and physiological responses in pigs, highlighting both the resilience and adaptability of the pigs’ immune system during periods of stress.

## Conclusion

In conclusion, we present evidence indicating that rearing environment and handling frequency influence growth and immune outcomes independently, but interactive effects were limited. Artificially-reared pigs displayed increased body weight gain prior to the group-housing context, thereby resulting in a higher body weight by study conclusion. This increased growth rate of artificially-reared pigs translated to increased small intestinal weights due to allometric organ growth. Additionally, fecal concentrations of secretory IgA were lower in artificially-reared pigs, possibly due to reduced pathogen exposure and limited intake of immunoglobulins. Artificially-rearing pigs maintained an upregulation of small intestinal pro-inflammatory cytokine expression two weeks after transitioning to the group-housing context, which may reflect a delayed or prolonged stress response. Handling frequency also influenced stress outcomes, as daily-handled pigs exhibited increased expression of pro-inflammatory cytokine and tight-junction proteins, which could be attributed to a more robust immune response from greater stress exposure at an earlier age, potentially indicating increased stress resiliency. However, further research is warranted to better understand the effects of rearing environment and handling frequency on stress responses and growth in young pigs.

## Abbreviations

ADFI: average daily feed intake
ADG: average daily gain
ANOVA: analysis of variance
AR-D: artificially-reared, daily-handled
AR-W: artificially-reared, weekly-handled
BW: body weight
CLDN: claudin
ELISA: enzyme linked immunosorbent assay
G: F: gain to feed
IFN-γ: interferon γ
IgA: immunoglobulin A
IL-1β: interleukin-1 β
LPS: lipopolysaccharide
OCLN: occludin
PND: postnatal day
SR-D: sow-reared, daily-handled
SR-W: sow-reared, weekly-handled
TNF-α: tumor necrosis factor-α
ZO-1: zonula occludens-1
